# The effects of local street network characteristics on the positional accuracy of automated geocoding for geographic health studies

**DOI:** 10.1186/1476-072X-9-10

**Published:** 2010-02-16

**Authors:** Dale L Zimmerman, Jie Li

**Affiliations:** 1 Department of Statistics and Actuarial Science, University of Iowa, Iowa City, IA 52242, USA; 2 Department of Biostatistics, University of Iowa, Iowa City, IA 52242, USA; 3 Center for Health Policy and Research, University of Iowa, Iowa City, IA 52242, USA

## Abstract

**Background:**

Automated geocoding of patient addresses for the purpose of conducting spatial epidemiologic studies results in positional errors. It is well documented that errors tend to be larger in rural areas than in cities, but possible effects of local characteristics of the street network, such as street intersection density and street length, on errors have not yet been documented. Our study quantifies effects of these local street network characteristics on the means and the entire probability distributions of positional errors, using regression methods and tolerance intervals/regions, for more than 6000 geocoded patient addresses from an Iowa county.

**Results:**

Positional errors were determined for 6376 addresses in Carroll County, Iowa, as the vector difference between each 100%-matched automated geocode and its ground-truthed location. Mean positional error magnitude was inversely related to proximate street intersection density. This effect was statistically significant for both rural and municipal addresses, but more so for the former. Also, the effect of street segment length on geocoding accuracy was statistically significant for municipal, but not rural, addresses; for municipal addresses mean error magnitude increased with length.

**Conclusion:**

Local street network characteristics may have statistically significant effects on geocoding accuracy in some places, but not others. Even in those locales where their effects are statistically significant, street network characteristics may explain a relatively small portion of the variability among geocoding errors. It appears that additional factors besides rurality and local street network characteristics affect accuracy in general.

## Background

Spatial epidemiologic studies commonly include statistical analyses of the spatial locations of study participants' residential addresses in order to, for example, test for geographic clustering of disease or estimate relationships between environmental exposures and disease [1,2]. Consequently, as part of the study's data assimilation process, the address provided by each study participant must be converted to geographic (e.g. latitude-longitude) coordinates, a procedure which is known as *geocoding*. In some studies, geocoding is performed by visiting each address with a global positioning (GPS) receiver or by referencing a very accurate (e.g., orthophoto-rectified) image map; however, it is cheaper and hence much more common to obtain geocodes by an automated procedure, which uses widely available GIS software to match each address to a street segment georeferenced within a street database (e.g., a U.S. Census Bureau TIGER file) and then linearly interpolate the position of the address along that segment. This procedure, herein called *automated geocoding*, is also known as *street geocoding*. Alternative procedures, such as parcel geocoding and "rooftop" (address-point) geocoding are growing in use, but in the United States, at least, they are not yet as prevalent as street geocoding. Furthermore, parcel geocoding typically has much lower address match rates than street geocoding [3].

Unfortunately, the geocodes obtained by any procedure contain *positional errors*, defined as (vector) differences from the locations of addresses ascertained by geocoding to their corresponding true locations. Thus every geocoding procedure has associated with it some level of inaccuracy. Some procedures, however, are more inaccurate than others; in particular, automated geocoding is much more inaccurate than geocoding via GPS receivers or image maps. Several recent investigations have demonstrated that automated geocoding frequently results in positional errors of several hundred meters or more [4-15]. For example, one study [7] in a four-county area of upstate New York found that 10% of a sample of rural addresses geocoded with errors of more than 1.5 km, and 5% geocoded with errors exceeding 2.8 km.

Zandbergen [16] lists four main components of *positional errors *associated with automated geocoding. First, the address may be assigned to the wrong street segment, due to errors in the input address fields or the street database. This typically results in very large positional errors. Second, the address may be assigned to the correct street segment, but the geographic coordinates of the entire segment in the street database are incorrect (e.g., shifted 100 m to the east). Together with the first component, this second component highlights the importance of using an accurate street database, a point emphasized recently in [17]. Third, the interpolated assignment of the address along the (correct) street segment may not coincide with the actual location of the address, due either to usage of only a portion of the segment's nominal address range or to less than perfect correspondence between a linear house numbering scheme and the actual numbering scheme on that segment, or both. Finally, the default offset (usually a uniform perpendicular distance of 10 to 15 m) used in automated geocoding may not accurately reflect the actual distance of the residence from the street centerline.

Positional errors introduce location uncertainties into the data that may affect spatial analytic methods. Documented effects of positional errors on spatial statistical analyses include an inflation of standard errors of parameter estimates and a reduction in power to detect spatial clusters and trends [18-22]. In order to better relate the size of these effects to the degree of automated geocoding inaccuracy, it is important to know how accuracy is affected by various geographic characteristics of an address. Such knowledge and understanding may make it possible, for example, to put context-specific confidence bounds or tolerance bounds on the magnitude of an address's positional error. Furthermore, they may allow one to simulate more realistic, context-specific positional errors for use in studies of the effects of geocoding inaccuracy on the power of various statistical tests for clusters, spatial trends, and other important spatial patterns and features; see, for example, [23]. Finally, it can facilitate and improve measurement-error model methods and imputation methods for adjusting spatial statistical analyses for geocoding inaccuracy [24-26].

Our current level of understanding of the geographic factors affecting automated geocoding accuracy is rather limited, however. One factor known to be important is whether an address lies in a rural or urban area. Every published study that has compared positional errors for rural and urban addresses within the same geographic region has found that automated geocodes of the former are, on average, not as accurate as those of the latter. The ratio (rural to urban) of mean positional error magnitudes has been variously reported as approximately 1.4:1 for a small study in western New York [6], 4:1 for a study spanning 49 states [12], 3:1 or 10:1 (depending on whether the automated geocoding was performed in-house or by a commercial firm) for a study in south central Iowa [11], 5:1 for a study in upstate New York [7], and 5:1 for the address data (from western Iowa) presented in this article. Another factor known to affect positional errors, at least in rural areas with strongly rectilinear street networks, is the axial orientation (north-south or east-west) of the street on which an address lies [15]; specifically, the directional error component in the direction aligned with the street tends to be greater than the component in the orthogonal direction. Presumably, this is due to errors of interpolation along the street segment that are larger, on average, than errors of offset from the segment. Furthermore, scatterplots of positional errors displayed in [7,12,15] and formal statistical analysis reported in [15] have revealed that the empirical probability distribution of positional errors is approximated poorly by a single bivariate normal distribution but quite well by a two-component or three-component mixture of bivariate normal or t distributions. The reason these mixtures fit better appears to be the fact that they can account for disparate components of errors (e.g. interpolation and offset errors) having considerably different levels of variability [15].

Notwithstanding what has been learned about automated geocoding's positional errors from previous studies, there is still much that is not well understood. For instance, there are readily available covariates in addition to rurality and orientation that may be associated with automated geocoding accuracy. Among these are local characteristics of the street network, for example street intersection density or street segment length. Street intersection density could be construed as a more refined measure of rurality than the dichotomous rural/urban classification measure used heretofore. As such, we might expect that in rural areas at least, accuracy would increase with an increase in street intersection density. Street segment length might be suspected of being associated with accuracy because of how address interpolation algorithms work. That is, since a linear interpolation algorithm places an address proportionately along a street segment, according to where the residence number falls in the range of street numbers assigned to the segment's endpoints, it is reasonable to expect the magnitudes of positional errors to be approximately proportional to segment length.

In this article we present analyses of the effects of several factors, including local characteristics of the street network, on automated geocoding accuracy. In our analyses we consider not only how means of positional error magnitudes are affected by such characteristics, but also, more comprehensively, how the entire distribution of positional error magnitudes is so affected. We use confidence intervals to characterize uncertainty associated with estimating mean positional error magnitudes, but for characterizing uncertainty associated with estimating the distribution of error magnitudes we use *tolerance intervals*, i.e. intervals that contain, with a given level of certainty, a fixed proportion of the error magnitudes. Tolerance intervals have a long history of use in engineering and the physical sciences for the purpose of quantifying the uncertainty of errors associated with manufacturing and other physical processes, and it is entirely reasonable to apply them, for the same purpose, to errors incurred by geocoding. Furthermore, to characterize uncertainty associated with estimating the bivariate distribution of positional error vectors, we construct tolerance regions.

The main purpose of this article is to investigate the relationship between automated geocoding accuracy and various geographic and street network characteristics, namely rurality, street orientation, street intersection density, and street segment length for a real data set of geocoded addresses. For this purpose, we use a rather large set of geocoded addresses from an Iowa county.

## Methods

### Iowa data

The address data upon which this investigation is based are a subset of all 9298 residential addresses in Carroll County, Iowa, USA, current as of 31 December 2005, which we obtained in conjunction with a comprehensive study of rural health in Iowa by the Iowa Department of Public Health and other researchers at the University of Iowa.

**Figure 1:**
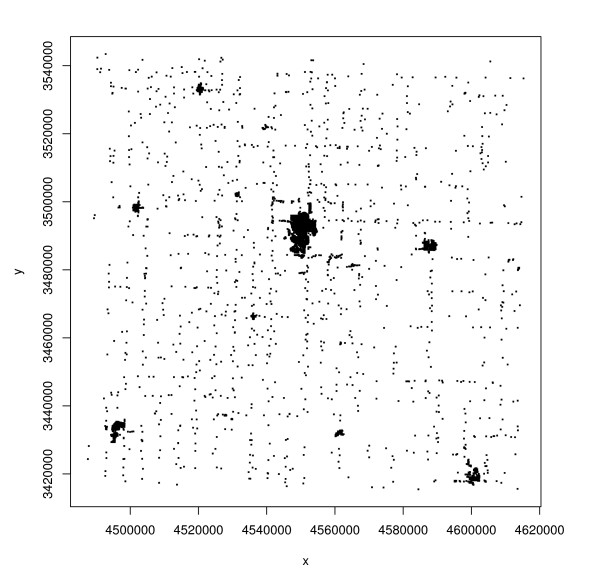
**Spatial locations of 6376 geocoded addresses in Carroll County, Iowa**.

**Figure 2:**
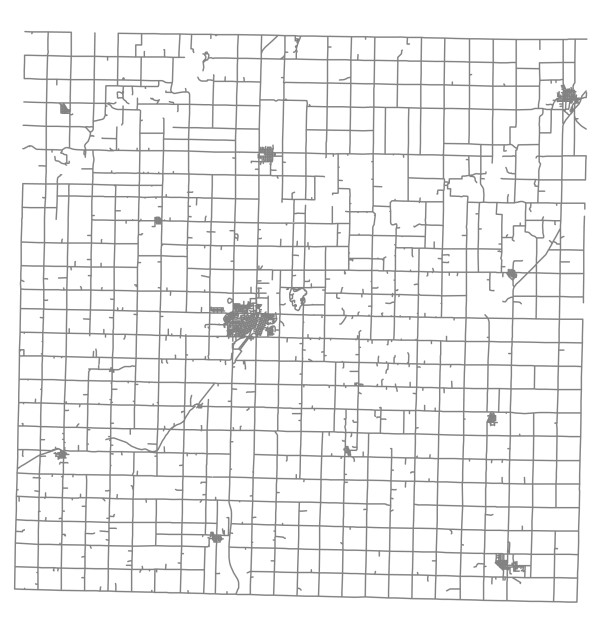
**Street map of Carroll County, Iowa**.

An automated geocoding procedure was performed for each address using ArcGIS 9.1. In addition, rural addresses were geocoded using an "orthophoto method," and municipal addresses were geocoded by an "E-911 method." Specifics of each method are described in [15], so we do not repeat them here. Because the orthophoto method is extremely accurate, rural geocodes obtained by this method were taken as the "gold standard" or truth. For municipal addresses, orthophoto geocodes were not available; however, the E-911 method is much more accurate than the automated method and we therefore regarded the E-911 geocode of a municipal address as the address's true location. Thus the positional error of the automated geocode of a rural address was determined as the difference between the automated and orthophoto geocodes, while that of a municipal address was determined as the difference between the automated and E-911 geocodes. Note that these positional errors are two-dimensional vectors, having an east-west component and a north-south component. We refer to the norm of this vector, or equivalently the Euclidean distance between the automated and ground-truthed (orthophoto or E-911) geocodes, as the *positional error magnitude*. We limited our set of addresses to those for which an automated geocode could be obtained using a 100%-match criterion and for which the orthophoto-derived geocode (for rural addresses) or E-911 geocode (for municipal addresses) could be ascertained unambiguously. This resulted in a dataset of 6376 addresses, of which 1421 (22%) were rural and 4955 (78%) were municipal. Ground-truthed locations of these addresses are displayed in Figure 1. The corresponding street map is given by Figure 2.

Corresponding to each address, the following covariates were measured: (1) a dichotomous rurality variable (rural or municipal); (2) a dichotomous street segment orientation variable (north-south or east-west); (3) street segment length; and (4) street intersection density. Street segment lengths were calculated automatically using ArcGIS's field calculator function and VBScript code available from ArcGIS help files. Street intersection density for a given address was measured by counting the number of intersections in a circular buffer of radius one mile centered on the address.

### Statistical methods

We investigated possible effects of street network characteristics on geocoding accuracy using classical regression methods. First, scatterplots of positional error magnitudes against values of each continuous covariate of interest (e.g. street segment length or street intersection density) were plotted, and the strength of the linear association between these variables was summarized by Pearson's correlation coefficient, *r*. Next, to describe more precisely how a change in a covariate affects positional accuracy, we fitted various regression models by ordinary least squares. The simplest model we considered was given by(1)

where *y *represents the magnitude of a positional error, or some transformation thereof; *x *represents the covariate of interest (e.g. street intersection density or street segment length); *α *and *β *are the *y*-intercept and slope, respectively, of an assumed straight line relating the expectation of *y *to *x*; and *e *represents model error. In accordance with fitting the model by ordinary least squares and subsequent normal theory-based inference, we assumed that the model errors are independent and identically distributed as normal random variables with mean zero and unknown variance *σ*^2^. We also considered larger, multiple regression models similar to (1), but which included one or more of the dichotomous covariates and interactions among them.

Within the context of the simple linear regression models, we obtained confidence intervals for the mean positional error magnitude, and upper tolerance bounds for a specified proportion of the error magnitudes, at selected values of the covariates. The standard two-sided 100(1 - *α*)% confidence interval for the expected value of *y *at a given *x *in this setting is given by(2)

where  and  are the ordinary least squares estimates of *α *and *β*, *t*_*α*/2,*n*-2 _is the 100(1 - *α*/2)th percentile of a t distribution with *n *- 2 degrees of freedom, *s*^2 ^is the mean squared error from the regression,

and  is the average of the *x*_*i*_'s. Under the same model, an upper 100(1 - *α*)% tolerance bound for the lower 100(1 - *p*)% of the population of positional error magnitudes for addresses with covariate equal to *x *is given by an expression of the same form as the upper 100(1 - *α*)% confidence limit in (2) except that *t*_*α*/2,*n *- 2 _is replaced with *t*_*α*,*n *- 2,*δ*(*x*)_, the 100(1 - *α*)th percentile of a noncentral t distribution with *n *- 2 degrees of freedom and noncentrality parameter *δ*(*x*) [27]. Here *δ*(*x*) = *z*_*p*_/*c*(*x*) and *z*_*p *_is the 100(1 - *p*)th percentile of the standard normal distribution.

We also obtained two-dimensional tolerance *regions *for the positional errors themselves (rather than their magnitudes). A 100(1 - *α*)% tolerance region for 100(1 - *p*)% of the distribution of positional errors is a region *R *such that the probability is 1 - *α *that *R *contains at least 100(1 - *p*)% of the positional errors in the population. Specifically, we obtained two approximate 95% tolerance regions for an inner 95% of the positional error distribution, both of which are ellipses. The first such region, the classical one due to John [28], is based on an assumption of bivariate normality for the positional error distribution, and is given by the set of (*y*_1_, *y*_2_)-values for which(3)

Here  is the centroid of positional errors, **S **is the sample covariance matrix of positional errors,  is the 100(1 - *α*)th percentile of the chi-square distribution with 2(*n *- 1) degrees of freedom, and  is the 100(1 - *p*)th percentile of the noncentral chi-square distribution with 2 degrees of freedom and noncentrality parameter 2/*n*. The second tolerance region, due to Di Bucchianico et al. [29], is the minimum volume ellipse containing  of the observed positional errors, where *z*_*α *_is the 100(1 - *α*)th percentile of the standard normal distribution and [·] is the greatest integer function. This tolerance region is nonparametric, i.e. distribution-free, meaning that it is valid regardless of the actual bivariate distribution of the positional errors. For the sample sizes of subgroups occurring in this study (which all exceed 600), exact determination of minimum volume ellipses (and hence the desired tolerance regions) was computationally prohibitive, so we determined them approximately via the resampling algorithm of Rousseeuw and Van Zomeren [30].

Although the proposed tolerance intervals for error magnitudes account for the effects of street segment length and street intersection density, neither tolerance region for the errors themselves does. To the authors' knowledge, multi-dimensional tolerance regions that condition on the values of continuous covariates such as these are not yet available. However, we do obtain separate tolerance regions for each of the four subgroups formed by the two categories of rurality and the two categories of street orientation.

## Results and Discussion

### Descriptive statistics

**Figure 3:**
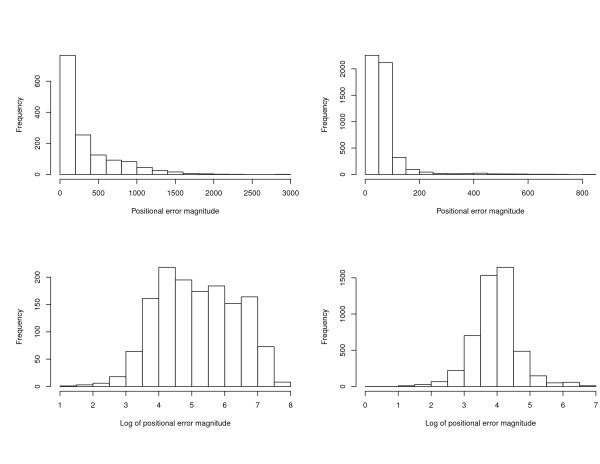
**Histograms of positional error magnitudes (in meters) for the automated geocodes of Carroll County addresses**. Upper left panel: error magnitudes for rural addresses. Upper right panel: error magnitudes for municipal addresses. Lower left panel: natural log of error magnitudes for rural addresses. Lower right panel: natural log of error magnitudes for municipal addresses.

Histograms of the positional error magnitudes are displayed in Figure 3, and summary statistics are given in Table 1. The top panels of Figure 3 indicate that the distribution of positional error magnitudes is strongly skewed to the right. These error magnitudes range from a minimum of 1 m to a maximum of 2896 m, with mean 127 m and median 59 m. As expected, there is a substantial difference between mean error magnitudes of rural and municipal addresses. The mean and median error magnitudes of rural addresses are 333 m and 168 m, which are approximately five and three times larger, respectively, than the mean and median error magnitudes of municipal addresses (68 m and 53 m). Standard deviations of rural and municipal error magnitudes appear to be roughly proportional to their means, suggesting that a log transformation of magnitude will make the empirical distributions more normal. Zandbergen [31] also found this to be so for positional error magnitudes corresponding to addresses from Orange County and Volusia County, Florida, USA. The histograms in the bottom panels of Figure 3 confirm that the logs of the positional error magnitudes for our Carroll County addresses are approximately normally distributed.

**Table 1:** Descriptive statistics for positional error magnitudes and directional displacement magnitudes of automated geocodes of Carroll County addresses.

Addresses	Sample size	Minimum	Median	Maximum	Mean	Standard deviation
All	6376	1	59	2896	127	219
Rural PEM	1421	3	168	2896	333	379
Rural |Δ*x*|, N-S	760	0	69	763	102	111
Rural |Δ*y*|, N-S	760	0	120	2892	318	420
Rural |Δ*x*|, E-W	661	0	59	1870	247	352
Rural |Δ*y*|, E-W	661	0	55	770	90	101
Municipal PEM	4955	1	53	839	68	71
Municipal |Δ*x*|, N-S	1812	0	24	354	26	28
Municipal |Δ*y*|, N-S	1812	0	42	839	55	62
Municipal |Δ*x*|, E-W	1735	0	43	700	63	88
Municipal |Δ*y*|, E-W	1735	0	22	632	25	26

Units for all descriptive statistics are meters.

**Figure 4:**
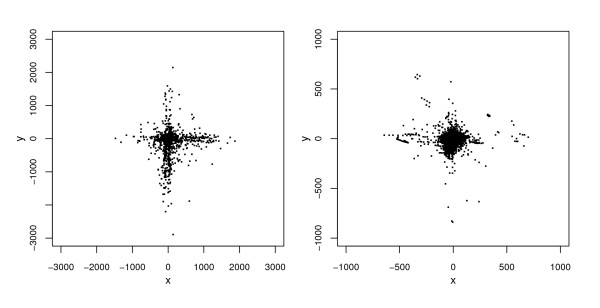
**Scatterplots of positional errors (in meters) for the automated geocodes of Carroll County addresses**. Left panel: rural addresses. Right panel: municipal addresses.

Plots of the bivariate distribution of errors are displayed in Figure 4. These indicate that the distribution of rural errors differs from that of municipal errors with respect to more than just mean error magnitude. In particular, rural errors (left panel) tend to cluster along the N-S and E-W axial directions in such a way that the overall shape of their distribution, apart from a few outliers, resembles a Greek cross. More rural errors lie near the center of the cross than near its extremities. Municipal errors (right panel) also exhibit some clustering along the two coordinate axes, but this clustering appears relatively less pronounced, as more errors are concentrated around the origin and the outliers are relatively larger and more numerous than for rural errors.

**Figure 5:**
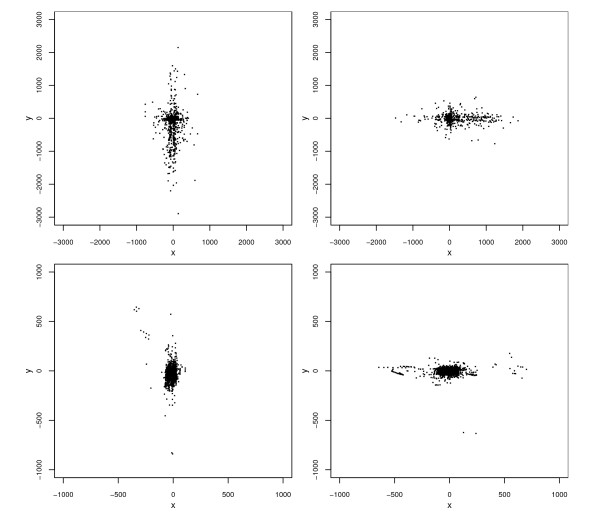
**Scatterplots of positional errors (in meters) for the automated geocodes of Carroll County addresses, by rurality and axial orientation of the street on which the corresponding address lies**. Upper left panel: rural address on N-S streets. Upper right panel: rural addresses on E-W streets. Lower left panel: municipal addresses on N-S streets. Lower right panel: municipal addresses on E-W streets.

In order to further investigate the clustering of positional errors along axes, we plotted positional errors for addresses on streets running mainly N-S separately from errors for addresses on streets running mainly E-W (Figure 5). We also obtained summary statistics separately for the directional error components of addresses for streets running in each of these two directions (Table 1). Among rural addresses there were 761 on N-S streets and 662 on E-W streets. The corresponding counts among municipal addresses were 1812 and 1735; in addition there were 1409 municipal addresses on mainly "diagonal" (neither N-S nor E-W) streets, which were omitted from Figure 5. The plots reveal that for both rural and municipal addresses, a positional error tends, as expected, to be aligned with the axial orientation of the street on which the address lies. This was also true for the omitted municipal addresses on diagonal streets (plot not included), which goes a long way toward explaining why the clustering along the N-S and E-W axes in Figure 4 is less pronounced for municipal addresses. Thus, axial street orientation appears to be a very important covariate for explaining the relative magnitudes of an address's N-S or E-W error components. Specifically, displacement along a street segment is, on average, two to three times larger than displacement perpendicular to the segment.

### Covariate effects

First, to characterize the proportion of variation in log positional error magnitude attributable to the effect of rurality, we carried out a one-factor analysis of variance. We found the effect of rurality to be highly significant (*P *< 2.0 × 10^-16^), but it explains only 28% of the overall variation in log positional error magnitude.

**Figure 6:**
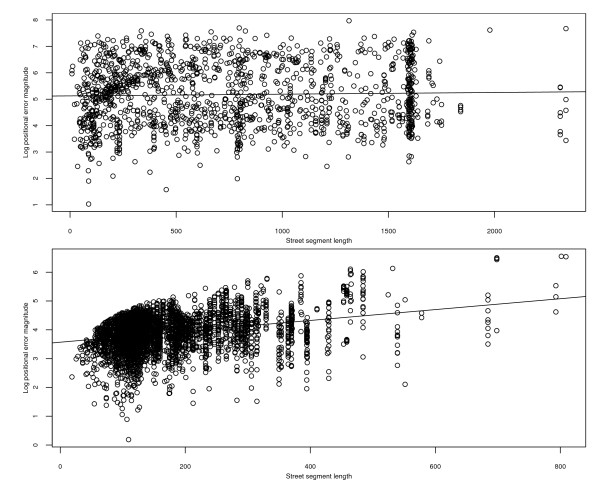
**Scatterplots of positional error magnitudes (on natural log scale) versus street segment length for the automated geocodes of Carroll County addresses**. Top panel: rural addresses. Bottom panel: municipal addresses. The superimposed lines are the fitted ordinary least squares regression lines. Units of street segment length and error magnitudes are meters.

Next we considered the question of whether the magnitude of an address's positional error is associated with either the length of the street segment on which the address lies or the street intersection density in the vicinity of the address (or both). A scatterplot of the natural logarithms of error magnitudes versus street segment length for rural addresses (Figure 6, top panel) indicates, somewhat surprisingly, that there is no association between these two variables (*r *= 0.03, *P *> 0.20). In contrast, the analogous plot for municipal addresses (Figure 6, bottom panel) reveals a noisy (*r *= 0.29) but nevertheless highly statistically significant (*P *< 2.0 × 10^-16^) positive association between these two variables. There is no curvature evident in the plot, suggesting that, apart from substantial noise, logs of municipal positional error magnitudes are linearly associated with street segment length. The least squares regression line (superimposed on the plot) indicates that the estimated mean positional error magnitude for municipal addresses on a 100-meter street segment, say, is 43 meters, and that this mean increases by about 21% with every 100-meter increase in the length of the street segment.

**Figure 7:**
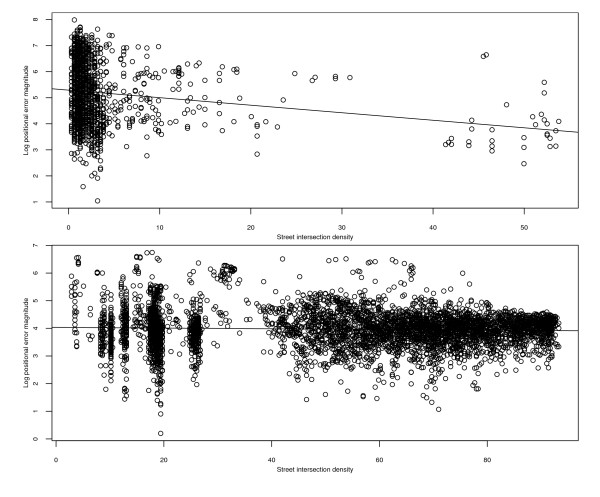
**Scatterplots of positional error magnitudes (on natural log scale) versus street intersection density for the automated geocodes of Carroll County addresses**. Top panel: rural addresses. Bottom panel: municipal addresses. The superimposed lines are the fitted ordinary least squares regression lines. Units of street intersection density are intersections per square mile, and units of error magnitudes are meters.

Figure 7 comprises two scatterplots, the top one for rural addresses and the bottom one for municipal addresses, of the natural logarithms of error magnitudes versus street intersection density. The top scatterplot indicates that log error magnitude and street intersection density are negatively associated for rural addresses (*r *= -0.19, *P *< 1.0 × 10^-11^), though there is considerable noise. Furthermore, the least squares regression line (superimposed on the plot) shows that the estimated mean log positional error magnitude for rural addresses with a street intersection density of, say, 5 intersections per square mile is 5.15 (172 meters), and that the mean error magnitude itself decreases by about 16% with every increase in intersection density of 5 intersections per square mile. The bottom scatterplot indicates that the association between these variables for municipal addresses is much weaker, though it is still statistically significant (*r *= -0.05, *P *= 0.0005).

We note that the correlation between street length and intersection density is -0.19 for rural addresses and -0.03 for municipal addresses -- values that are very similar to the correlations between error magnitude and intersection density. As a consequence, the partial correlations between error magnitude and either covariate, adjusted for the other covariate, are virtually identical to the corresponding ordinary correlations. That is, the relationships between error magnitude and either covariate, which were described in the previous two paragraphs, are not affected by whether we do or do not adjust for the values of the other covariate.

In an effort to obtain a model for the entire set of log positional error magnitudes (rural and municipal) with the greatest possible explanatory power, we also fitted a multiple linear regression model with covariates rurality, street length, and street intersection density and their two-way and three-way interactions. All coefficient estimates are highly significant. However, the overall *R*^2 ^for the model is only 0.31, which is not much larger than that for the model that includes only the effect of rurality. Thus, the degree of explanatory power for the model is disappointingly modest.

### Confidence limits and tolerance bounds

Next we computed confidence limits and tolerance bounds on the positional error magnitudes, based on simple linear regression models relating log positional error magnitude to the covariates that had the most highly statistically significant effects, as reported in the previous section: street length for the municipal data, and street intersection density for the rural data. For the municipal data only, we obtained 95% confidence intervals for the mean log positional error magnitude at the 10th, 50th, and 90th percentiles of street length. We also obtained the 95% tolerance bound for the lower 95% of log positional error magnitudes when street length is at its 10th, 50th, and 90th percentiles. These confidence limits and tolerance bounds were then exponentiated to express them in the original measurement scale (meters). Similarly, for the rural data only, we obtained 95% confidence intervals for the mean log positional error magnitude and the 95% tolerance bound for the lower 95% of log positional error magnitudes at the 10th, 50th, and 90th percentiles of street intersection density; and then exponentiated them. Results are given in Table 2. Since the 95% tolerance bound is an estimated upper bound for the 95th percentile of the conditional distribution of positional error magnitudes at a given value of the significant covariate, it is much larger than the 95% upper confidence limit for the mean of the same distribution. However, it behaves in much the same way: it increases with street length for the municipal data, and it decreases with street intersection density for the rural data.

**Table 2:** Two-sided 95% confidence limits for mean positional error magnitude, and 95% tolerance bound for the lower 95% of positional error magnitudes, at the 10th, 50th, and 90th percentiles of the statistically most important covariate (street length for municipal addresses and street intersection density for rural addresses).

Rurality	Covariate	*p*	100*p*th percentile	Confidence limits	Tolerance bound
Municipal	Length	0.10	93.7	(41.87,42.88)	111.26
		0.50	127.8	(44.73,45.75)	118.56
		0.90	295.4	(60.98,63.04)	163.17
Rural	Density	0.10	0.64	(180.49,210.00)	1436
		0.50	1.59	(176.08,203.71)	1395
		0.90	7.00	(150.07,174.84)	1195

Units of these limits and bounds are meters.

### Tolerance regions

**Figure 8:**
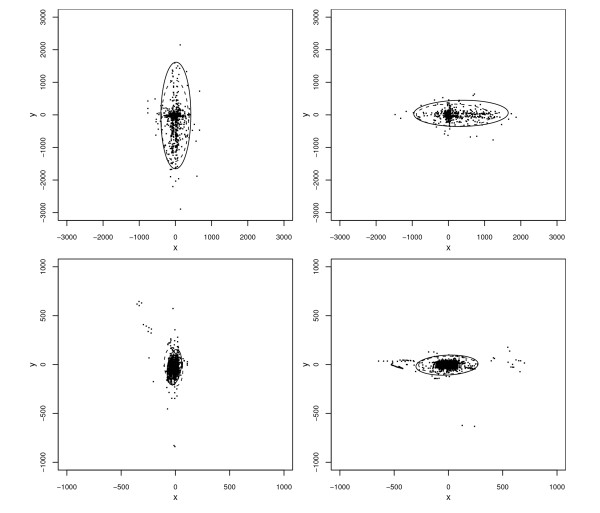
**Scatterplots of positional errors (in meters) for the automated geocodes of Carroll County addresses, by axial orientation of the street on which the corresponding address lies, with 95% normality-based tolerance regions for the inner 95% of the distributions**. Upper left panel: rural address on N-S streets. Upper right panel: rural addresses on E-W streets. Lower left panel: municipal addresses on N-S streets. Lower right panel: municipal addresses on E-W streets. In all panels, the outer boundary of the normality-based and nonparametric elliptical tolerance regions are represented by solid and dashed curves, respectively.

Finally, we computed tolerance regions for 95% of the bivariate distribution of positional errors. More specifically, for each combination of rurality and orientation, we obtained 95% elliptical tolerance regions for an inner 95% of the distribution of positional errors. Both the normality-based region and the nonparametric region described previously were obtained. Results are displayed in Figure 8. Normality-based tolerance regions for municipal addresses are seen to be smaller than those for rural addresses, but rurality appears to have almost no effect on the orientation of the region's major axis, which is always aligned with street orientation apart from possibly a slight tilt. Nor does rurality appear to affect the ratio of the elliptical region's major axis length to its minor axis length. For three of the four groups of addresses, the nonparametric region is slightly larger than its normality-based counterpart. The exception is the group of municipal north-south addresses, for which the normality-based region appears to be dilated by the large concentration of extreme outliers in the upper left of the plot, which causes the sample covariance matrix to be considerably larger than it would otherwise be.

## Conclusions

The major issue this study addressed is what the effects of certain street network characteristics may be on the accuracy of automated geocoding of patient addresses. For the Carroll County address data, we found that:

• Mean positional errors for rural addresses are about five times larger, and more strongly clustered in the E-W and N-S axial directions, than their municipal counterparts.

• The effect of street segment length on geocoding accuracy was statistically significant for municipal addresses, for which, as expected, mean error magnitude increased with length. There was no such effect for rural addresses, however. We note that a similar phenomenon -- a significant positive street length effect for municipal but not rural addresses -- was observed for another, much smaller dataset of 95 addresses (54 municipal, 41 rural) of cancer patients from Kentucky (Eric Durbin, personal communication). This suggests that this phenomenon may not be uncommon, but more evidence is needed to substantiate this.

• The effect of proximate street intersection density on geocoding accuracy was statistically significant for rural addresses, and as expected this effect was such that mean error magnitude was inversely related to intersection density. For municipal addresses, this inverse relationship was also found to be statistically significant, but it was of much smaller magnitude.

• Although the effects of one or more street network characteristics were found to be statistically significant, unfortunately they explained only a modest proportion of the variability in the positional errors, especially when considered in the context of a model that accounts for rurality. Thus, the utility of street length and street intersection density as predictors of geocoding accuracy appears to be limited. It is possible that local street network characteristics other than the ones we considered contribute more to geocoding accuracy. Additional measurable factors that could be studied include the nominal address range for a street segment (some segments have much larger ranges than others) and the ratio of actual address range (maximum house number minus minimum house number) to nominal address range for a segment. Further work is needed to determine whether these or other measurable covariates affect accuracy.

Carroll County's strongly rectilinear road network, its relatively high rural/municipal population ratio, and its lack of a truly urban area are typical of many counties in the midwestern region of the United States, but not of many counties in other regions. Thus, the extent to which results for other areas would be similar to those for Carroll County is unknown. We hope that others will perform similar investigations using address data from regions of the United States or other countries with less rectilinear road networks and larger urban areas.

In addition to investigating the significance of street network characteristic effects, we applied methodology for obtaining confidence intervals and tolerance intervals for positional error magnitudes of the Carroll County addresses, which take into account the values of local street network characteristics. For the Carroll County positional error vectors themselves, we obtained elliptical tolerance regions, both parametric (normality-based) and nonparametric, which accounted for rurality and street orientation. Despite the relatively small proportion of the overall variability explained by the covariates, accounting for them in the computation of tolerance intervals and regions does appear to be worthwhile, as they facilitate a more address-specific assessment of likely positional error than is possible when the covariates are ignored.

In focusing our attention on geocoding errors, we have ignored the fact that for many studies, automated geocoding is incomplete; that is, not all addresses can be assigned point-level spatial coordinates by the software. In fact, it is common in practice for 20% or even as many as 40% of subjects' addresses to fail to geocode using standard software and street files. For example, Gregorio et al. [32] and Oliver et al. [33] present public health studies in which 14% and 26%, respectively, of addresses could not be assigned a point location via automated geocoding. For the Carroll County addresses of the present study this figure was 20% (36% rural, 15% municipal) under a 60%-match criterion (and slightly higher under a 100%-match criterion). Possible effects of street network characteristics on the failure to geocode is a topic for future study.

Finally, we note that our study focused on *global *effects of street network characteristics on geocoding accuracy. Alternatively, one could allow these effects to be spatially varying and use methods of local modeling such as geographically weighted regression [34] to characterize their spatial variation.

## Competing interests

The authors declare that they have no competing interests.

## Authors' contributions

DLZ conceived of this study and wrote the majority of the manuscript. JL performed most of the statistical analyses. Both authors read and approved the final manuscript.
